# Avaliação da Disfunção Endotelial em Casos de COVID-19 com Dilatação Fluxo-Mediada

**DOI:** 10.36660/abc.20210561

**Published:** 2022-05-24

**Authors:** Asli Kurtar Mansiroglu, Hande Seymen, Isa Sincer, Yilmaz Gunes

**Affiliations:** 1 Abant Izzet Baysal University Hospital Departamento de Cardiologia Bolu Turquia Abant Izzet Baysal University Hospital, Departamento de Cardiologia, Bolu – Turquia

**Keywords:** COVID-19/complicações, Células Endoteliais/infecção;, Endotélio Vascular/lesões, Diagnóstico por Imagem/métodos, Ecocardiografia/métodos, Ultrassonografia/métodos, Dilatação do Fluxo Mediado, Mialgia, Distúrbios do Olfato, Distúrbios do Paladar

## Abstract

**Fundamento:**

Sabe-se que a inflamação desempenha um papel crucial em muitas doenças, incluindo a COVID-19.

**Objetivo:**

Utilizando a dilatação fluxo-mediada (DFM), objetivou-se avaliar os efeitos da inflamação na função endotelial de pacientes com COVID-19.

**Métodos:**

Este estudo foi realizado com um total de 161 indivíduos, dos quais 80 foram diagnosticados com COVID-19 nos últimos seis meses (48 mulheres e 32 homens com idade média de 32,10±5,87 anos) e 81 eram controles saudáveis (45 mulheres e 36 homens com idade média de 30,51±7,33 anos). Os achados do ecocardiograma transtorácico e da DFM foram analisados em todos os indivíduos. Resultados com p<0,05 foram considerados estatisticamente significantes.

**Resultados:**

O ecocardiograma e a DFM do grupo COVID-19 foram realizados 35 dias (intervalo: 25–178) após o diagnóstico. Não houve diferença estatisticamente significativa nos parâmetros ecocardiográficos. Em contraste, a DFM (%) foi significativamente maior no grupo controle (9,52±5,98 versus 12,01±6,18; p=0,01). Na análise multivariada com o modelo *stepwise* progressivo, a DFM foi significativamente diferente no grupo controle em relação ao grupo COVID-19 (1,086 (1,026–1,149), p=0,04). O teste de correlação de Spearman indicou que a DFM (r=0,27; p=0,006) apresentou correlação positiva fraca com a presença de COVID-19.

**Conclusão:**

Os achados deste estudo apontam para disfunção endotelial induzida por COVID-19, avaliada por DFM, na fase inicial de recuperação.

## Introdução

Um novo tipo de doença causada por coronavírus surgiu em dezembro de 2019, sendo chamada de COVID-19 pela Organização Mundial da Saúde (OMS). Ela infecta principalmente o trato respiratório e se espalhou rapidamente pelo mundo.^1^

Como vírus de RNA capazes de sofrer mutações e se recombinar rapidamente, os coronavírus são conhecidos por infectar sobretudo o trato respiratório ou o trato intestinal em humanos e animais.^2^ Os coronavírus entram na célula hospedeira ligando-se à enzima conversora de angiotensina 2 da peptidase de zinco, uma molécula de superfície encontrada nas células endoteliais de artérias e vasos, no epitélio do trato respiratório, no músculo liso arterial, no epitélio do intestino delgado e em células imunes.^3-5^

A ativação e a disfunção endotelial se desenvolvem como resultado da infeção das células endoteliais pela COVID-19.^6^ Isso aumenta os níveis de citocinas pró-inflamatórias (fator de necrose tumoral alfa, interleucina-1 e interleucina-6/IL-6), quimiocinas (proteína quimiotática de monócitos-1), antígeno do fator de von Willebrand (FvW), e atividade FvW, fator anti-hemofílico (FAH) e reagentes de fase aguda (IL-6, proteína C reativa e dímero D).^6^

Embora a COVID-19 afete principalmente os tratos respiratórios superior e inferior, o endotélio vascular é outro alvo conhecido. A disfunção endotelial pode ser causada diretamente pela atividade do vírus ou pela resposta inflamatória sistêmica resultante. A dilatação fluxo-mediada (DFM) — método ultrassonográfico não invasivo — tem sido bastante utilizada para avaliar a disfunção endotelial devido à sua simplicidade e economia.^7^ Vários estudos têm abordado o efeito da DFM em doenças inflamatórias, como artrite reumatoide, doença vascular periférica, doença arterial coronariana, diabetes mellitus (DM) e hipertensão. Até o momento e até onde sabemos, há apenas alguns relatos sobre o uso da DFM para avaliar a COVID-19.^8,9^

Neste estudo, a DFM foi utilizada para investigar possíveis efeitos anormais na função vascular de pacientes recuperados de uma infecção de COVID-19.

## Métodos

Este estudo de centro único foi realizado no Abant Izzet Baysal University Training and Research Hospital entre outubro de 2020 e fevereiro de 2021. A pesquisa contou com a participação de 80 indivíduos diagnosticados com COVID-19 nos últimos seis meses que não necessitaram de hospitalização e 81 indivíduos controle saudáveis, com distribuição etária de >18 e <45 anos. Todos os pacientes do grupo COVID-19 estavam curados e sem sintomas no momento da entrada no estudo.

Os critérios de exclusão foram: idade >45 anos, qualquer presença de doença arterial coronariana, disfunção sistólica do ventrículo esquerdo (fração de ejeção/FE<50%), valvopatia moderada a grave, cardiopatia congênita, distúrbio de condução atrioventricular, doença renal ou hepática moderada a grave, doença tireoidiana, desequilíbrio eletrolítico, doença inflamatória sistêmica ou janela acústica ecocardiográfica inadequada. O protocolo do estudo foi aprovado pelo Comitê de Ética local e um termo de consentimento livre e esclarecido foi assinado por cada indivíduo antes da participação.

Com base no Plano de Diagnóstico e Tratamento de COVID-19 da Comissão Nacional de Saúde (7ᵃ edição), os casos de COVID-19 foram classificados em quatro tipos clínicos: leves (sintomas clínicos leves sem pneumonia em imagem radiológica), comuns (febre, comprometimento do trato respiratório e outros sintomas com pneumonia em imagem radiológica), graves (desconforto respiratório, frequência respiratória de ≥30 vezes/min, saturação de oxigênio ≤93% em repouso, PaO2/FiO2 ≤300 mmHg) e críticos (insuficiência respiratória com necessidade de ventilação mecânica, choque e insuficiência de outro órgão exigindo monitoramento e tratamento em uma unidade de terapia intensiva).^10^

O comprometimento pulmonar foi categorizado por meio do “escore total de gravidade” (ETG), baseado na avaliação da tomografia computadorizada (TC) de tórax. Para isso, as porcentagens de comprometimento calculadas para cada um dos cinco lobos foram convertidas em uma das seguintes categorias de pontuação: nenhum (0%) (escore 0), mínimo (1–25%) (escore 1), leve (26–50%) (escore 2), moderado (51–75%) (escore 3) e grave (76–100%) (escore 4). Por fim, a soma de todas as pontuações gerou um valor de ETG que variou de 0 a 20.^11^

Parâmetros laboratoriais foram obtidos a partir de prontuários hospitalares no diagnóstico da infeção de COVID-19. Os dados laboratoriais do grupo controle foram coletados na entrada do estudo.

Pacientes e controles foram avaliados por meio de ecocardiograma e ultrassonografia braquial com Doppler para obter a medida da DFM na entrada do estudo.

### Avaliação ecocardiográfica

Um transdutor Vivid S6 de 4 MHz (GE Vingmed, N-3191 Horten-Noruega) foi utilizado para realizar os procedimentos ecocardiográficos necessários.

Todas as imagens ecocardiográficas foram obtidas por meio do monitoramento contínuo do eletrocardiograma (ECG) por um cardiologista com cegamento simples com os pacientes na posição lateral esquerda. Considerou-se a média de três ciclos cardíacos consecutivos e foram medidos os diâmetros diastólico final e sistólico final do ventrículo esquerdo, a espessura da parede posterior do ventrículo esquerdo, a espessura do septo ventricular esquerdo e os diâmetros do átrio esquerdo. O método de Simpson biplano modificado foi aplicado para medir a FE do ventrículo esquerdo. Medidas Doppler bidimensionais e pulsadas foram calculadas com base nos critérios da American Society of Echocardiography.^12^

### Avaliação ultrassonográfica

Os parâmetros foram obtidos em uma sala silenciosa, escura e com ar-condicionado (ou seja, temperatura ambiente de 22–25°C) após um período de repouso de pelo menos 15 minutos. Além disso, foi solicitado aos participantes que evitassem se exercitar, fumar e consumir álcool ou cafeína por pelo menos 8 horas antes das medições de DFM. Um transdutor de arranjo linear de 7,5 MHz (GE Healthcare, M4S-RS, Tóquio, Hino-Shi, Japão) foi empregado para medir o diâmetro da artéria braquial na fossa antecubital. A pele foi marcada com um lápis; portanto, todas as medidas foram realizadas na mesma linha. Iniciou-se com o diâmetro basal e a taxa de fluxo da artéria braquial e, em seguida, a pressão foi aumentada até 50 mmHg acima da pressão arterial sistólica e mantida por 5 minutos nesse nível, de modo que o braço permanecesse isquêmico. Em seguida, a pressão do balonete foi reduzida, e o diâmetro e a taxa de fluxo da artéria braquial foram medidos novamente após 1 minuto da queda da pressão.

A DFM foi calculada usando a seguinte equação:


$$DFM\;=100\text{× }(di\hat{a}metro\;máximo\;no\;1^{\circ }\;minuto\;-\;di\hat{a}metro\;de\;refer\hat{e}ncia)/di\hat{a}metro\;de\;refer\hat{e}ncia.^{13}$$

### Análise estatística

Todas as análises estatísticas foram realizadas por meio do SPSS 18.0 Statistical Package Software para Windows (SPSS Inc., Chicago, IL, EUA). Os dados de normalidade das variáveis foram avaliados pelo teste de Kolmogorov-Smirnov. Variáveis contínuas com distribuição normal foram descritas por média e desvio padrão; variáveis contínuas sem distribuição normal foram descritas por mediana e intervalo interquartil (IIQ). Os dados foram expressos em números ou porcentagens para variáveis qualitativas. Para analisar as diferenças entre grupos independentes, utilizou-se o teste *t* de Student (bicaudal) para variáveis quantitativas com distribuição normal, o teste U de Mann-Whitney para variáveis sem distribuição normal e o teste qui-quadrado para variáveis qualitativas. Análises de correlação de Spearman foram realizadas para avaliar correlações entre a COVID-19 e o nível de linfócitos, a relação neutrófilos/linfócitos, os níveis de glicose e creatinina e a DFM. Para as variáveis consideradas significativas na análise de regressão univariada, empregou-se a regressão logística multivariada com o modelo *stepwise* progressivo para estabelecer os fatores prognósticos independentes da COVID-19. O teste de correlação de Spearman também foi realizado entre a DFM e o tempo decorrido desde o diagnóstico. Resultados com p<0,05 foram considerados estatisticamente significantes.

## Resultados

Características clínicas de referência foram semelhantes entre os dois grupos. Entre os parâmetros laboratoriais, glicose, creatinina e a relação neutrófilos/linfócitos foram significativamente maiores, enquanto as contagens de linfócitos foram significativamente menores no grupo COVID-19 quando comparadas ao grupo controle (Tabela 1).

**Tabela 1:** Variáveis demográficas e laboratoriais da população do estudo

Variáveis			COVID-19 (n= 80)	Grupo controle (n=81)	p
**Dados demográficos**					
Idade (anos)			32,10±5,87	30,51±7,33	0,407
Homens/Mulheres (n (%))			32/48 (40/60%)	36/45 (44/56%)	0,313
PAS (mmHg) (IIQ)			105 (14)	110 (22)	0,307
PAD (mmHg) (IIQ)			70 (15)	70 (20)	0,343
Altura (cm)			169,36±8,72	169,36±9,30	0,997
Peso (kg)			73,81±13,73	71,30±16,09	0,289
IMC (kg/m^2^)			25,63±3,74	25,00±4,13	0,198
Hipertensão n (%)		Não	78 (97,5%)	79 (97,5%)	1,000
		Sim	2 (2,5%)	2 (2,5%)	
Diabetes mellitus n (%)		Não	78 (97,5%)	81 (100,0%)	0,245
		Sim	2 (2,5%)	0 (0,0%)	
Dislipidemia n (%)		Não	79 (98,8%)	80 (98,8%)	1,000
		Sim	1 (1,3%)	1 (1,2%)	
Histórico familiar de DAC n (%)		Não	54 (67,5%)	59 (72,8%)	0,459
		Sim	26 (32,5%)	22 (27,2%)	
Fumante n (%)		Não	61 (76,3%)	58 (48,7%)	0,502
		Sim	19 (23,8%)	23 (28,4%)	
**Parâmetros laboratoriais**					
	Glicose plasmática em jejum (mg/dL) (IIQ)		93,50 (16,75)	91 (14)	0,038
	Creatinina (mg/dL) (IIQ)		0,78 (0,19)	0,74 (0,11)	0,042
	Hemoglobina (g/dL) (IIQ)		14,20 (2,05)	14,40 (1,95)	0,875
	Hematócrito (%) (IIQ)		42,30 (5,51)	42,90 (5,40)	0,851
	Contagem de plaquetas (K/uL) (IIQ)		250 (90,25)	263 (76,50)	0,659
	Contagem de linfócitos (K/uL) (IIQ)		1,83 (1,14)	2,24 (0,89)	0,017
	Contagem de neutrófilos (K/uL) (IIQ)		4,15 (2,12)	3,82 (1,73)	0,291
	Relação neutrófilos/linfócitos (IIQ)		2,05 (1,63)	1,30 (1,06)	0,044

*IIQ: intervalo interquartil; PAS: pressão arterial sistólica; PAD: pressão arterial diastólica; IMC: índice de massa corporal; DAC: doença arterial coronariana.

Mialgia (65%) e perda de olfato e/ou paladar (61%) foram os sintomas mais comuns em pacientes com COVID-19, enquanto sudorese (8%) foi o menos comum (Tabela 2). Nenhum dos pacientes do grupo COVID-19 apresentou infeção grave que exigisse hospitalização. Neste estudo, os pacientes tinham o tipo leve ou o comum de acordo com a classificação clínica, com ETG variando de 0 a 5.

**Tabela 2:** Sintomas dos pacientes que tiveram COVID-19

Sintomas	Número	%
Mialgia	52/80	65
Perda de olfato e/ou paladar	49/80	61
Fraqueza	33/80	41
Dor de cabeça	32/80	40
Tosse	28/80	35
Febre	25/80	31
Dispneia	19/80	24
Dor de garganta	15/80	19
Náusea	15/80	19
Diarreia	13/80	16
Sudorese	7/80	8

O ecocardiograma e a DFM do grupo COVID-19 foram realizados 35 dias (25–178; IIQ: 38,5) após o diagnóstico. As medidas ecocardiográficas foram semelhantes entre os dois grupos. No entanto, em relação ao grupo controle, a DFM (%) foi significativamente menor nos pacientes com COVID-19 (9,52±5,98 versus 12,01±6,18, p=0,010) (Tabela 3). O teste de correlação de Spearman mostrou não haver associação estatisticamente significante entre a DFM e o tempo decorrido desde o diagnóstico de COVID-19 (r=0,064; p=0,527).

**Tabela 3:** Medidas ecocardiográficas da população do estudo

Variáveis	COVID-19 (n= 80)	Grupo controle (n= 81)	p
Diâmetro do átrio esquerdo (cm)	3,03±0,5	2,92±0,32	0,332
DDFVE (cm)	4,48±0,45	4,45±0,42	0,281
DSFVE (cm)	2,80±0,30	2,81±0,29	0,711
PP (cm)	0,96±0,14	0,96±0,13	0,550
SIV (cm)	0,92±0,16	0,90±0,14	0,742
FE (%)	67,27±5,02	65,90±4,64	0,151
Onda E transmitral (cm/s) (IIQ)	96,9 (23,3)	94,7 (22,5)	0,409
Onda A transmitral (cm/s) (IIQ)	68,0 (16,1)	69,0 (15,3)	0,533
TD mitral (ms) (IIQ)	198 (45)	188 (57)	0,531
E' lateral (cm/s) (IIQ)	12,2 (3)	12,5 (3,5)	0,414
A' lateral (cm/s) (IIQ)	9,35 (2,5)	9,0 (3)	0,515
S' lateral (cm/s) (IIQ)	9,5 (2)	10,0 (2,1)	0,066
TAPSE (cm) (IIQ)	2,19 (0,44)	2,16 (0,40)	0,537
PSAP (mmHg)	23,79±5,13	25,14±5,63	0,268
DFM (%)	9,52±5,98	12,01±6,18	0,010

*DDFVE: diâmetro diastólico final do ventrículo esquerdo; DSFVE: diâmetro sistólico final do ventrículo esquerdo; PP: parede posterior; SIV: septo interventricular; FE: fração de ejeção; IIQ: intervalo interquartil; TD: tempo de desaceleração; E': pico de velocidade diastólica inicial do tecido miocárdico; A': pico de velocidade diastólica final do tecido miocárdico; S': velocidade miocárdica sistólica do anel mitral; DFM: dilatação fluxo-mediada; TAPSE: excursão sistólica do plano do anel tricúspide; PSAB: pressão sistólica da artéria pulmonar.

Parâmetros significativamente diferentes na análise de regressão univariada (glicose, creatinina, linfócitos, relação neutrófilos/linfócitos e DFM) foram incluídos na análise de regressão multivariada e apenas o valor da DFM foi significativamente diferente no grupo controle em relação ao grupo COVID-19 (1,086 (1,026–1,149), p=0,04) (Tabela 4).

**Tabela 4:** Preditores independentes de COVID-19 na análise de regressão logística multivariada

	OR (IC95%)	p
Glicose	0,981 (0,957–1,005)	0,116
Linfócitos	1,022 (0,646–1,616)	0,926
Relação neutrófilos/linfócitos	0,895 (0,744–1,077)	0,240
Creatinina	0,093 (0,005–1,595)	0,101
DFM	1,086 (1,026–1,149)	0,004

DFM: dilatação fluxo-mediada; IC: intervalo de confiança; OR (odds ratio): razão de chances.

O teste de correlação de Spearman indicou que a DFM (r=0,27, p=0,006) apresentou correlação positiva fraca com a presença de COVID-19.

## Discussão

O objetivo deste trabalho foi avaliar as repercussões vasculares da COVID-19, utilizando a DFM comprometida como marcador substituto da disfunção endotelial. Este estudo demonstrou que o valor da DFM foi menor em pacientes do grupo COVID-19 em comparação ao grupo controle. Os resultados apontam para um comprometimento vascular por COVID-19, avaliado pela DFM, mesmo em pacientes com quadros leves. Até onde sabemos, este é o primeiro estudo a determinar a disfunção endotelial vascular por DFM comprometida entre pacientes jovens que se recuperam de uma infeção leve de COVID-19.

Foi detectada uma redução significativa na DFM mesmo em pacientes pouco afetados logo após a recuperação. Isso levanta a questão sobre a possibilidade de a doença ter efeitos anormais de longo prazo na função vascular. Achados semelhantes foram reportados por Ergul et al.,^8^ que avaliaram 63 pacientes que tiveram COVID-19 dois meses após a recuperação e identificaram a infeção por COVID-19 e o aumento do índice de massa corporal como preditores independentes de disfunção endotelial avaliados pela DFM.^8^

Da mesma forma, Riou et al.,^9^ encontraram uma redução significativa na DFM de 16 pacientes com COVID-19 leve a moderada, enquanto a DFM tendia a ser menor em nove pacientes com COVID-19 grave a crítica três meses após o início da doença.^9^ Ao contrário desses relatos, o presente estudo se concentrou em pacientes com COVID-19 leve, não hospitalizados, 35 dias (25–178) após o início da doença.

Sabe-se que a disfunção endotelial, associada ao estresse oxidativo, é o primeiro fator de muitas doenças.^14^ Embora a inflamação faça parte da resposta natural de recuperação do corpo à cura e seja essencial para proteger o corpo contra infeções e substâncias ambientais perigosas, seria bastante otimista dizer que é completamente benéfica. Quando ela sai do controle, pode se tornar prejudicial e destrutiva para o corpo.^15^ A inflamação sistemicamente fora de controle também está associada a desfechos adversos da COVID-19.^16^

Em um estudo em que a DFM foi usada para predizer eventos cardiovasculares futuros em pacientes submetidos à cirurgia de revascularização miocárdica, a menor taxa de evento foi encontrada em pacientes com DFM normal (>8%), enquanto a taxa de evento moderada e a maior taxa de evento foram detectadas em pacientes com DFM de 4 a 8% e <4%, respetivamente.^17^ Em outra pesquisa, pacientes com DFM inferior a 6,2% obtiveram um índice tornozelo-braquial significativamente menor em relação aos com DFM superior a 6,2%.^18^ Além disso, Maruhashi et al.,^19^ demonstraram que a DFM apresentou correlação inversa com o Escore de Risco de Framingham, comumente usado como calculadora de risco e índice de risco cardiovascular cumulativo para avaliar a probabilidade de ataque cardíaco ou morte por cardiopatia dentro de 10 anos.^19^

Fatores independentes preditivos de mortalidade por COVID-19 incluem idade avançada, comorbidades como diabetes mellitus (DM), doença cardiovascular, câncer e doença pulmonar obstrutiva crônica na apresentação.^20^ Entretanto, bebês e crianças não tiveram aumento significativo tanto em morbidade quanto em mortalidade durante a pandemia de COVID-19.^21^

Com o aumento da idade e das doenças relacionadas à idade, o estado inflamatório crônico torna-se dominante e a resposta anti-inflamatória do sistema imunológico torna-se irregular e incapaz de suprimir o episódio inflamatório de forma rápida e eficaz.^22^ O presente estudo buscou excluir os efeitos de inflamações relacionadas à idade mais avançada, incluindo indivíduos com menos de 45 anos de idade.

Embora ainda dentro dos limites da normalidade, pacientes do grupo COVID-19 apresentaram níveis significativamente mais elevados de glicose e creatinina do que os do grupo controle.

Durante a fase aguda da infeção, os níveis de glicemia podem aumentar de forma anormal em pacientes sob estresse da COVID-19, mesmo que não tenham diagnóstico de DM. Também há relatos de impactos anormais na função renal. Altos níveis de glicemia em pacientes com COVID-19 podem ser preditivos de piores desfechos, independentemente do histórico de DM.^23^ A doença renal está associada ao aumento de mortalidade por COVID-19.^24^ Verificou-se que 14,4% de 701 pacientes hospitalizados com COVID-19 apresentaram aumento nos níveis séricos de creatinina, 13,1% tiveram redução na taxa de filtração glomerular e aproximadamente 5% apresentaram insuficiência renal aguda.^24^ Resultados histopatológicos revelaram lesões tubulares agudas, diferentes comprometimentos glomerulares, necrose tubular e glomeruloesclerose.^25^ Neste estudo, o achado de níveis levemente aumentados de glicemia e creatinina pode ser incidental, mas também pode sugerir lesão renal subclínica e/ou estresse contínuo.

A linfopenia tem sido usada no diagnóstico de COVID-19 e associada a um pior prognóstico.^26^ A gravidade da COVID-19 também foi correlacionada com a relação neutrófilos/linfócitos e a relação linfócitos/proteína C reativa.^27^ Assim, em comparação com o grupo controle, as contagens de linfócitos diminuíram e a relação neutrófilos/linfócitos aumentou nos participantes do estudo que tiveram COVID-19 leve.

### Limitações

As principais limitações desta pesquisa são a sua realização em centro único e o número relativamente pequeno de pacientes. Os resultados são limitados a um momento inicial do processo da doença e não podem ser extrapolados para refletir achados de longo prazo. Outra limitação é o fato de os parâmetros laboratoriais não terem sido aferidos simultaneamente com a medição da DFM. Devido aos critérios de exclusão e ao limite de idade, a população do estudo foi estritamente selecionada; portanto, os achados podem não representar todos os pacientes com COVID-19.

## Conclusão

Este estudo mostrou uma diminuição da DFM em pacientes jovens com quadro leve de COVID-19 na fase inicial de recuperação. Assim, esse parâmetro pode ser usado como marcador para disfunção endotelial induzida pela COVID-19. Sem dúvida, o monitoramento cardiovascular de rotina em pacientes com histórico de COVID-19 pode identificar indivíduos com risco de eventos cardiovasculares futuros. Para entender melhor os possíveis efeitos cardiovasculares nesses pacientes, deve-se considerar estudos de larga escala, incluindo seguimento a longo prazo.
