# Supplementary material for: Avaliação da Disfunção Endotelial em Casos de COVID-19 com Dilatação Fluxo-Mediada
Source: Arq Bras Cardiol. 2022 May 24;119(2):319–25. [Article in Portuguese] doi: 10.36660/abc.20210561 (PMC9363072; doi:10.36660/abc.20210561)
Supplement: Translation

# Translation: Evaluation of Endothelial Dysfunction in COVID-19 With Flow-Mediated Dilatation

**Authors:** Asli Kurtar Mansiroglu, Hande Seymen, Isa Sincer, Yilmaz Gunes

**Affiliations:** 1 Bolu Turkey Abant Izzet Baysal University Hospital, Departamento de Cardiologia, Bolu – Turkey

**Keywords:** COVID-19/complications, Endothelial, Cells/infection, Endothelium Vascular/injuries, Diagnostic Imaging/methods, Echocardiography/methods, Ultrasonography/methods, Flow Dilatation, Myalgia, Olfaction Disorders, Taste Disorders

## Abstract

**Background:**

Inflammation is known to play a crucial role in many diseases, including COVID-19.

**Objective:**

Using flow-mediated dilatation (FMD), we aimed to assess the effects of inflammation on endothelial function in COVID-19 patients.

**Methods:**

This study was conducted with a total of 161 subjects, of whom 80 were diagnosed with COVID-19 within the last six months (comprising 48 women and 32 men with a mean age of 32.10 ± 5.87 years) and 81 were healthy controls (comprising 45 women and 36 men with a mean age of 30.51 ± 7.33 years). We analyzed the findings of transthoracic echocardiography and FMD in all subjects. All results were considered statistically significant at the level of p < 0.05.

**Results:**

The echocardiography and FMD of the COVID-19 group were performed 35 days (range: 25-178) after diagnosis. There was no statistically significant difference in echocardiographic parameters. Differently, FMD (%) was significantly higher in the control group (9.52 ± 5.98 vs. 12.01 ± 6.18, p=0.01). In multivariate analysis with the forward stepwise model, FMD was significantly different in the control group compared to the COVID-19 group (1.086 (1.026 - 1.149), p=0.04). A Spearman’s correlation test indicated that FMD (r=0.27, p=0.006) had a weak positive correlation with the presence of COVID-19.

**Conclusion:**

Our findings point to COVID-19-induced endothelial dysfunction, as assessed by FMD, in the early recovery phase.

## Introduction

A new type of coronavirus disease emerged in December 2019 and was named COVID-19 by the WHO. It primarily infects the respiratory tract and has spread rapidly around the world.^1^

As RNA viruses that can rapidly mutate and recombine, coronaviruses are known to primarily infect the respiratory tract or intestinal tract in humans and animals.^2^ Coronaviruses enter the host cell by binding to the zinc peptidase angiotensin-converting enzyme 2, a surface molecule found in the endothelial cells of arteries and vessels, the respiratory tract epithelium, the arterial smooth muscle, the small intestinal epithelium, and immune cells.^3-5^

Endothelial activation and dysfunction develop as a result of endothelial cells being infected with COVID-19.^6^ They lead to increased levels of pro-inflammatory cytokines (tumor necrosis factor-alpha, interleukin-1, and interleukin-6), chemokines (monocyte chemoattractant protein-1), von Willebrand factor (vWF) antigen, vWF activity, anti-hemophilic factor (AHF), and acute-phase reactants (IL-6, C-reactive protein, and D-dimer).^6^

Although COVID-19 primarily affects the upper and lower respiratory tracts, the vascular endothelium is another known target. Endothelial dysfunction may be caused directly by the activity of the virus or by the resulting systemic inflammatory response. Flow-mediated dilatation (FMD), which is a non-invasive ultrasonographic method, has been widely used to evaluate endothelial dysfunction due to its simplicity and cost-efficiency.^7^ Several studies have addressed the effect of FMD on various inflammatory diseases such as rheumatoid arthritis, peripheral vascular disease, coronary artery disease, diabetes mellitus, and hypertension. To date, as far as we know, there are only a few reports on FMD being used to evaluate COVID-19.^8,9^

In this study, we used FMD to investigate the potential abnormal effects of COVID-19 on the vascular function of patients recovered from a COVID-19 infection.

## Methods

This single-centered study was carried out at Abant Izzet Baysal University Training and Research Hospital between October 2020 and February 2021. The study included 80 subjects diagnosed with COVID-19 within the last six months who did not require hospitalization and 81 healthy control subjects, with an age distribution of > 18 and < 45 years. All the COVID-19 patients were cured and free from symptoms at the time of study entry.

The exclusion criteria were as follows: age > 45 years, any presence of coronary artery disease, left ventricle systolic dysfunction (EF < 50%), moderate to severe valvular disease, congenital heart disease, atrioventricular conduction abnormality, moderate to severe kidney or liver disease, thyroid disease, electrolytic imbalance, systemic inflammatory disease, or poor acoustic echocardiography window. The study protocol was approved by the Local Ethics Committee and a written informed consent form was signed by each subject before participation.

Based on the COVID-19 Diagnosis and Treatment Plan by the National Health Commission (7^th^ edition), COVID-19 cases were classified into four clinical types: mild (characterized by mild clinical symptoms without pneumonia on radiological imaging), common (characterized by fever, involvement of the respiratory tract, and other symptoms with pneumonia on radiological imaging), severe (characterized by respiratory distress, respiratory rate of ≥ 30 times/min, oxygen saturation ≤ 93% at rest, PaO2/FiO2 ≤ 300 mmHg), and critical (characterized by respiratory failure requiring mechanical ventilation, shock, and failure of another organ requiring monitoring and treatment at an intensive care unit).^10^

Lung involvement was classified using the “total severity score” (TSS) based on an assessment of chest computed tomography (CT) imaging. For this purpose, the percentages of involvement calculated for each of the five lobes were converted into one of the following score categories: none (0%) (Score 0), minimal (1-25%) (Score 1), mild (26-50%) (Score 2), moderate (51-75%) (Score 3), and severe (76-100%) (Score 4). Finally, the sum of all the scores yielded a TSS value ranging from 0 to 20.^11^

Laboratory parameters were obtained from hospital medical records at COVID-19 infection diagnosis. Laboratory data from the control group were obtained at study entry.

Patients and control subjects were evaluated with echocardiography and brachial Doppler ultrasonography for FMD measurement at study entry.

### Echocardiographic evaluation

We used a Vivid S6 4-Mhz transducer (GE Vingmed, N-3191 Horten-Norway) to perform the required echocardiographic procedures.

All echocardiographic images were obtained using continuous ECG monitoring by a single-blind cardiologist with the subjects in the left lateral position. We considered the mean of three consecutive cardiac cycles and measured left ventricular end-diastolic and end-systolic diameters, left ventricular posterior wall thickness, left ventricular septum thickness, and left atrium diameters. A biplane modfied simpson’s method was applied for measuring left ventricular ejection fraction. We performed two-dimensional and pulsed Doppler measurements based on the American Society of Echocardiography criteria^12^.

### Ultrasonographic evaluation

The parameters were measured in a quiet, dark, and air-conditioned room (i.e. room temperature of 22 - 25°C) after a rest period of at least 15 minutes. In addition, subjects were asked to fast and to avoid exercising, smoking, and consuming alcohol or caffeine for at least 8 hours before FMD measurements. We used a 7.5 MHz linear array transducer (GE Healthcare, M4S-RS, Tokyo, Hino-Shi, Japan) to measure the brachial artery diameter at the antecubital fossa. The skin was marked with a pencil, and thus all measurements were performed on the same line. We started with the basal diameter and flow rate of the brachial artery and then increased the pressure up to 50 mmHg above systolic blood pressure, and waited for 5 minutes at this level, so the arm remained ischemic. Then cuff pressure was lowered, and the diameter and flow rate of the brachial artery were measured again at 1 minute after pressure decrease.

FMD was calculated using the following equation:


$$FMD\;=\;100\;\times \;(maximum\;diameter\;at\;the\;1^{st}\;minute-baseline\;diameter)/baseline\;diameter.^{13}$$

### Statistical analysis

All statistical analyses were performed using SPSS 18.0 Statistical Package Software for Windows (SPSS Inc., Chicago, IL, USA). Normality data of the variables were evaluated with the Kolmogorov-Smirnov test. Continuous variables with normal distribution were described using the mean and standard deviation; continuous variables without normal distribution were described using the median and interquartile range. The data are shown as numbers or percentages for qualitative variables. To analyze differences between independent groups, we used the Student’s t-test (two-tailed) for normally distributed quantitative variables, the Mann-Whitney’s U-test for variables without normal distribution, and the Chi-square test for qualitative variables. Spearman’s correlation analyses were conducted to evaluate correlations between COVID-19 and lymphocyte level, neutrophil/lymphocyte ratio, glucose and creatinine levels, and FMD. For variables found to be significant in the univariate regression analysis, we employed multivariate logistic regression with the forward stepwise model to establish the independent prognostic factors of COVID-19. Spearman’s correlation test was also performed between FMD and time elapsed from diagnosis. All results were considered statistically significant at the level of p < 0.05.

## Results

Baseline clinical characteristics were similar between both groups. Among laboratory parameters, glucose, creatinine, and neutrophil/lymphocyte ratio were significantly higher; lymphocyte counts were significantly lower in the COVID-19 group compared to the control group (Table 1).

**Table 1:** Demographic and laboratory variables of the study population

Variables			COVID-19 (n= 80)	Control Group (n=81)	p
**Demographics**					
Age (years)			32.10±5.87	30.51±7.33	0.407
Male/Female (n(%))			32/48 (40/60%)	36/45 (44/56%)	0.313
SBP (mmHg) (IIQ)			105 (14)	110 (22)	0.307
DBP (mmHg) (IIQ)			70 (15)	70 (20)	0.343
Height (cm)			169.36±8.72	169.36±9.30	0.997
Weight (kg)			73.81±13.73	71.30±16.09	0.289
BMI (kg/m^2^)			25.63±3.74	25.00±4.13	0.198
Hypertension n (%)		No	78 (97.5%)	79 (97.5%)	1.000
		Yes	2 (2.5%)	2 (2.5%)	
Diabetes mellitus n (%)		No	78 (97.5%)	81 (100.0%)	0.245
		Yes	2 (2.5%)	0 (0.0%)	
Hyperlipidemia n (%)		No	79 (98.8%)	80 (98.8%)	1.000
		Yes	1 (1.3%)	1 (1.2%)	
Family History of CAD n (%)		No	54 (67.5%)	59 (72.8%)	0.459
		Yes	26 (32.5%)	22 (27.2%)	
Smoking n (%)		No	61 (76.3%)	58 (48.7%)	0.502	
		Yes	19 (23.8%)	23 (28.4%)		
**Laboratory parameters**					
	Fasting Plasma Glucose (mg/dL) (IIQ)		93.50 (16.75)	91 (14)	0.038
	Creatinine (mg/dL) (IIQ)		0.78 (0.19)	0.74 (0.11)	0.042
	Hemoglobin (g/dL) (IIQ)		14.20 (2.05)	14.40 (1.95)	0.875
	Hematocrit (%) (IIQ)		42.30 (5.51)	42.90 (5.40)	0.851
	Platelet counts (K/uL) (IIQ)		250 (90.25)	263 (76.50)	0.659
	Lymphocyte counts (K/uL) (IIQ)		1.83 (1.14)	2.24 (0.89)	0.017
	Neutrophil counts (K/uL) (IIQ)		4.15 (2.12)	3.82 (1.73)	0.291
	Neutrophil/ lymphocyte ratio (IIQ)		2.05 (1.63)	1.30 (1.06)	0.044

*IIQ: Interquartile Range; SBP: systolic blood pressure; DBP: diastolic blood pressure; BMI: body mass index.

Myalgia (65 %) and loss of smell and/or taste (61 %) were the most common symptoms in COVID-19 patients, whereas sweating was (8 %) the least common one (Table 2). None of the COVID-19 patients had a serious infection that required hospitalization. In our study, the patients belonged either to the mild type or the common type, according to the clinical classification, with TSS scores ranging from 0 to 5.

**Table 2:** Symptoms experienced by the COVID-19 patients

Symptoms	Number	%
Myalgia	52/80	65
Loss of smell and/or taste	49/80	61
Weakness	33/80	41
Headache	32/80	40
Cough	28/80	35
Fever	25/80	31
Dyspnea	19/80	24
Sore throat	15/80	19
Nausea	15/80	19
Diarrhea	13/80	16
Sweating	7/80	8

Echocardiography and FMD of the COVID-19 group were performed 35 days (25-178; IIQ: 38.5) after diagnosis. Echocardiographic measurements were similar between the two groups, whereas compared to the control group FMD (%), it was significantly lower in the COVID-19 patients (9.52 ± 5.98 vs. 12.01 ± 6.18, p=0.010) (Table 3). Spearman’s correlation test showed that there was no statistically significant relation between FMD and the time elapsed after COVID-19 diagnosis (r=0.064; p=0.527).

**Table 3:** Echocardiographic measurements of the study population

Variables	COVID-19 (n= 80)	Control Group (n= 81)	p
Left atrium diameter (cm)	3.03±0.5	2.92±0.32	0.332
LVDD (cm)	4.48±0.45	4.45±0.42	0.281
LVSD (cm)	2.80±0.30	2.81±0.29	0.711
PW (cm)	0.96±0.14	0.96±0.13	0.550
IVS (cm)	0.92±0.16	0.90±0.14	0.742
EF (%)	67.27±5.02	65.90±4.64	0.151
Transmitral E wave (cm/s) (IIQ)	96.9 (23.3)	94.7 (22.5)	0.409
Transmitral A wave (cm/s) (IIQ)	68.0 (16.1)	69.0 (15.3)	0.533
Mitral DT (ms) (IIQ)	198 (45)	188 (57)	0.531
Lateral E’ (cm/s) (IIQ)	12.2 (3)	12.5 (3.5)	0.414
Lateral A’ (cm/s) (IIQ)	9.35 (2.5)	9.0 (3)	0.515
Lateral S’ (cm/s) (IIQ)	9.5 (2)	10.0 (2.1)	0.066
TAPSE (cm) (IIQ)	2.19 (0.44)	2.16 (0.40)	0.537
sPAB (mmHg)	23.79±5.13	25.14±5.63	0.268
FMD (%)	9.52±5.98	12.01±6.18	0.010

*LVDD: left ventricular diastolic diameter; LVSD: Left ventricular systolic diameter; PW: posterior wall; IVS: interventricular septum; EF: Ejection fraction; IIQ: Interquartile Range; DT: deceleration time; E’: peak early diastolic myocardial tissue velocity; A’: peak late diastolic myocardial tissue velocity; S: mitral annular systolic myocardial velocity; FMD: Flow-mediated dilatation; TAPSE: tricuspid annular plane systolic excursion; sPAB: systolic pulmonary artery pressure.

Significantly different parameters in the univariate regression analysis (glucose, creatinine, lymphocyte, neutrophil/lymphocyte ratio, and FMD) were included in the multivariable regression analysis and only the FMD value was significantly different in the control group compared to the COVID-19 group (1.086 (1.026 – 1.149), p=0.04) (Table 4).

**Table 4:** Independent predictors of COVID-19 by multivariate logistic regression analysis

	OR (95%CI)	p
Glucose	0.981 (0.957–1.005)	0.116
Lymphocyte	1.022 (0.646–1.616)	0.926
Neutrophil/lymphocyte ratio	0.895 (0.744–1.077)	0.240
Creatinine	0.093 (0.005–1.595)	0.101
FMD	1.086 (1.026–1.149)	0.004

*FMD: flow-mediated dilatation; CI: Confidence interval; OR: Odds ratio.

Spearman’s correlation test showed that FMD (r=0.27, p=0.006) had a weak positive correlation with the presence of COVID-19.

## Discussion

The purpose of this study was to assess the vascular repercussions of COVID-19 using impaired FMD as a surrogate marker of endothelial dysfunction. Our study demonstrated that the FMD value was lower in COVID-19 patients compared to the control group. These results point to vascular involvement by COVID-19, as assessed by FMD, even in mildly affected patients. Within the limits of our knowledge, this is the first study to show vascular endothelial dysfunction defined by impaired FMD among young patients recovering from a mild COVID-19 infection.

We found a significant reduction in FMD even in such mildly affected patients early after recovery. This raises the question of whether the disease may have long-term abnormal effects on vascular function. Similarly to our findings, Ergul et al.^8^ included 63 COVID-19 patients two months after recovery and found COVID-19 infection and increased body mass index as independent predictors of endothelial dysfunction evaluated by FMD.^8^

Likewise, Riou et al.^9^ found a significant decrease in FMD among 16 mild-to-moderate COVID-19 patients, whereas FMD tended to be lower among 9 severe-to-critical COVID-19 patients three months after disease onset.^9^ Contrary to these reports, we studied mildly affected non-hospitalized COVID-19 patients 35 days (25-178) after disease onset.

Endothelial dysfunction, associated with oxidative stress, is known to be the earliest factor for many diseases.^14^ Although inflammation is part of the body’s normal repair response to healing and is essential in protecting our body from infections and dangerous environmental substances, it would be overly optimistic to say that it is completely beneficial. When it gets out of control, it can become detrimental and destructive to the body.^15^ Likewise, it is known that systemically out-of-control inflammation is associated with adverse COVID-19 outcomes.^16^

In a study where FMD was used to predict future cardiovascular events in patients who had undergone coronary bypass surgery, the lowest event rate was determined in patients with normal FMD (>8%), while a moderate event rate and the highest event rate were found in patients with an FMD value of 4 to 8% and <4%, respectively.^17^ In another study, patients with an FMD less than 6.2% had significantly lower ankle/brachial index compared to those with an FMD greater than 6.2%.^18^ In addition, Maruhashi et al.^19^ showed that FMD had an inverse correlation with the Framingham Risk Score, commonly used as a risk calculator and an index of cumulative cardiovascular risk for assessing the probability of a heart attack or death from heart disease within 10 years.^19^

Independent predictive factors of mortality from COVID-19 include advanced age, comorbidities such as diabetes mellitus (DM), cardiovascular disease or cancer, and chronic obstructive pulmonary disease at presentation.^20^ However, neither infants nor children showed a significant increase in both morbidity and mortality during the COVID-19 pandemic.^21^

With increased age and age-related diseases, the chronic inflammatory state becomes dominant, and the anti-inflammatory response of the immune system becomes erratic and unable to suppress the inflammatory episode in a timely and effective manner.^22^ In our study, we aimed to exclude the effects of such advanced age-related inflammation by including subjects under the age of 45 years.

Although still within the normal range, the COVID-19 patients had significantly slightly higher levels of blood glucose and creatinine than those of the control group. During the acute phase of infection, blood glucose levels may rise abnormally in patients under COVID-19 stress, even if they are not diagnosed with diabetes mellitus. Renal function has been also reported to be abnormally affected. High levels of blood glucose in COVID-19 patients can predict worse outcomes regardless of a DM history.^23^ Kidney disease is associated with increased mortality from COVID-19.^24^ It was found that 14.4% of 701 hospitalized patients with COVID-19 had increased serum creatinine levels, 13.1% had a decreased glomerular filtration rate, and approximately 5% had acute kidney injury.^24^ Histopathological findings revealed acute tubular injuries, different impairments of the glomeruli, tubular necrosis, and glomerulosclerosis.^25^ Our finding of slightly increased blood glucose and creatinine levels may be an incidental finding but also may suggest subclinical kidney injury and/or ongoing stress.

Lymphopenia has been used in the diagnosis of COVID-19 and has been associated with a poor prognosis.^26^ The severity of COVID-19 was also correlated with the neutrophil/lymphocyte ratio and the lymphocyte/CRP ratio.^27^ Accordingly, compared to the control group, lymphocyte counts were decreased and NLR was increased in our mild COVID-19 study subjects.

### Limitations

The main limitations of this study lie in the fact that it is single-centered and that it was conducted on a relatively small number of patients. The results are limited to an early point in time during the disease process and cannot be extrapolated to reflect long-term findings. Another limitation is that laboratory parameters were not measured simultaneously with FMD measurement. Due to the exclusion criteria and age limit, the study population was strictly selected, and therefore the results can not represent all COVID-19 patients.

## Conclusion

This study showed a decrease in FMD in young patients who were mildly affected by COVID-19 in the early recovery phase. Therefore, this parameter may be used as a marker for COVID-19-induced endothelial dysfunction. Undoubtedly, routine cardiovascular monitoring in patients with a history of COVID-19 may identify patients at risk of future cardiovascular events. To better understand the possible cardiovascular effects in these patients, larger-scale studies including long-term follow-up should be considered.
